# Impact of powered circular stapling devices on anastomotic leakage rates in colorectal surgery

**DOI:** 10.1007/s00384-026-05195-7

**Published:** 2026-07-08

**Authors:** Catherine Kollmann, Theresa Eckart, Beata Kusnezov, Lars Kollmann, Matthias Kelm, Christoph-Thomas Germer, Johan Friso Lock, Sven Flemming

**Affiliations:** Department of General, Visceral, Transplant, Vascular and Paediatric Surgery, University Hospital Würzburg, Oberdürrbacherstrasse 6, 97080 Würzburg, Germany

**Keywords:** Colorectal resection, Transanal anastomosis, Circular stapler, Anastomotic leakage, Manual stapler, Powered stapler

## Abstract

**Introduction:**

Anastomotic leakage (AL) remains a severe complication after colorectal surgery, increasing morbidity and mortality. Powered circular staplers may influence anastomotic healing through standardised compression and power distribution. This study aimed to compare clinical outcomes of powered versus manual circular staplers for transanal anastomoses.

**Method:**

This retrospective single-centre cohort study included consecutive patients undergoing colorectal resection with transanal circular anastomosis between January 2022 and June 2025. Primary endpoint was the incidence of AL according to stapler type. To ensure comparability between groups, propensity score matching was performed.

**Results:**

Among 333 patients, powered staplers were used in 69.1% and manual staplers in 30.9%. The overall AL rate was 15.0%. AL was significantly associated with higher BMI (28.1 vs. 26.6 kg/m²; p = .021), ASA ≥ 3 (64.0 vs. 46.0%; p = .019), diabetes mellitus (24.0 vs. 8.1%; p .001), open (34.0 vs. 19.1%) or converted surgeries (14.0 vs. 4.9%; p = .004) and greater intraoperative fluid administration (2548.1 vs. 2188.8 ml; p = .015). Most patients (72.1%) followed enhanced recovery concepts (FAST TRACK) with trend toward lower adherence in the AL cohort (n.s.).  After propensity score matching, AL occurred in 9.7% following powered circular stapling and 11.8% following manual circular stapling (n.s.).

**Conclusion:**

In this cohort, powered stapling devices were not associated with lower anastomotic leakage rates compared with manual staplers for transanal circular anastomosis in patients managed under modern minimally invasive and FAST TRACK concepts.

**Supplementary Information:**

The online version contains supplementary material available at 10.1007/s00384-026-05195-7.

## Introduction

Anastomotic leakage (AL) is the most relevant postoperative complication in colorectal surgery. It poses a major health burden by prolonging hospitalisation, increasing the need for additional interventions and raising healthcare costs, while also increasing patient morbidity, long-term ostomy dependence and mortality [1–3]. The incidence of AL estimates between 2.5 and 19.0% depending on various patient and surgical risk factors [1, 4, 5]. These predisposing risk factors for AL include e.g. male gender, obesity, advanced tumour stage or low-level anastomosis [1, 3, 5].

Since these factors lie beyond the surgeons’ control, current research focuses on modifiable, procedure-related determinants. Surgeon experience has been shown to improve postoperative outcomes and several procedures require a defined learning curve [6]. Advances in perioperative care, particularly through the promotion of enhanced recovery concepts (FAST TRACK), have further contributed to improved patient recovery [6].

Despite these developments, AL rates have remained largely unchanged. The elevated risk of AL in technically demanding anastomoses, such as rectal anastomoses, is thought to result from the constraints of the narrow pelvis, increased anastomotic tension and compromised microvascular perfusion [7]. However, the extent to which stapling technique and human factors contribute to AL development remains uncertain.

With the introduction of a powered stapler in 2019, it was hypothesized that improved force uniformity and motion stability might reduce AL risk. Nevertheless, current evidence is limited and characterised by contradictory findings [8–15].

In this study, we analysed the potential impact of a powered circular stapler on the AL rates in patients undergoing transanal anastomosis, in comparison with conventional manual staplers.

## Methods

### Study design and population

We conducted a retrospective single-centre cohort study of consecutive adult (≥ 18 years) patients undergoing colorectal resection with a primary transanal stapled anastomosis at University Hospital Würzburg during January 2022 and June 2025. This study is reported in accordance with the Strengthening the Reporting of Observational Studies in Epidemiology (STROBE) statement. A completed STROBE checklist is provided as Supplementary Table S1.

Included procedures were colectomy, left/extended left hemicolectomy, rectosigmoid resection and low anterior rectal resection with the creation of a primary transanal circular stapled anastomosis. Patients undergoing revisional surgery for chronic anastomotic leakage, discontinuity resections or hand-sewn anastomosis were excluded.

Demographical and clinical data were collected retrospectively; including age, sex, body mass index (BMI), comorbidities, medication, surgical details, stapler type and postoperative complications. The primary endpoint was AL. Secondary endpoints were length of stay, Comprehensive Complication Index (CCI) [16], Clavien-Dindo grade [17] of AL-related complications and mortality.

### Ethical approval

The Ethics Committee of the University of Würzburg approved the study (proposal number 20240919 01).

### Surgical procedures

Patients received open, laparoscopic or robotic intestinal resection with primary rectal or anal anastomosis. Anastomoses were created using 28–32 mm circular stapler, either manual (Covidien, Dublin, Ireland; Frankenman, Kiel, Germany) or powered (Ethicon, Hamburg, Germany). Protective ostomy was performed at the surgeon’s discretion.

### Diagnosis of anastomotic leakage

AL diagnosis followed our institutional FAST TRACK protocol. Supplementary Table S2 depicts our colorectal FAST TRACK protocol and Supplementary Figure S1 illustrates AL diagnostic algorithm [18]. Diagnostic evaluation was initiated after robot-assisted surgery for leucocytes ≥ 10.000/µl and/or C-reactive protein (CRP) > 20 mg/dl on postoperative day (POD) 3, or if leucocytes or CRP increased from POD 5 compared to POD 3. After laparoscopic-assisted or open surgery, diagnostics were performed for elevated leucocytes or CRP ≥ 20 mg/dl from POD 5 onward. Diagnostics were also triggered by clinical suspicion, such as fever, abdominal distension, peritonism or abnormal drain output. AL diagnostics included endoscopy followed by computed tomography (CT). AL was diagnosed when endoscopy showed insufficiency or fibrin-covered anastomosis and CT demonstrated leakage signs (perianastomotic collection, free air).

### Statistical analysis

Statistical analyses were performed using SPSS Statistics 29 (IBM Corporation, Armonk, NY, USA). Descriptive data are presented as means with 95% confidence intervals or frequencies. Categorical variables were compared using Pearson chi-square or Fisher’s exact test. Normality was tested by Shapiro–Wilk test. Parametric variables were analysed using the unpaired t-test or ANOVA with Welch correction, non-parametric variables were analysed using Mann–Whitney U or Kruskal–Wallis test. Propensity score matching (powered vs. manual stapler) based on age, sex, BMI, American Society of Anesthesiologists (ASA) classification, Charlson Comorbidity Index, diabetes, surgical approach and intraoperative crystalloid administration (caliper 0.01). Matching quality was assessed using standardised mean differences (Love plot, Supplementary Figure S2). Statistical significance was defined as *p* < 0.05.

## Results

### Patient characteristics

**Fig. 1:**
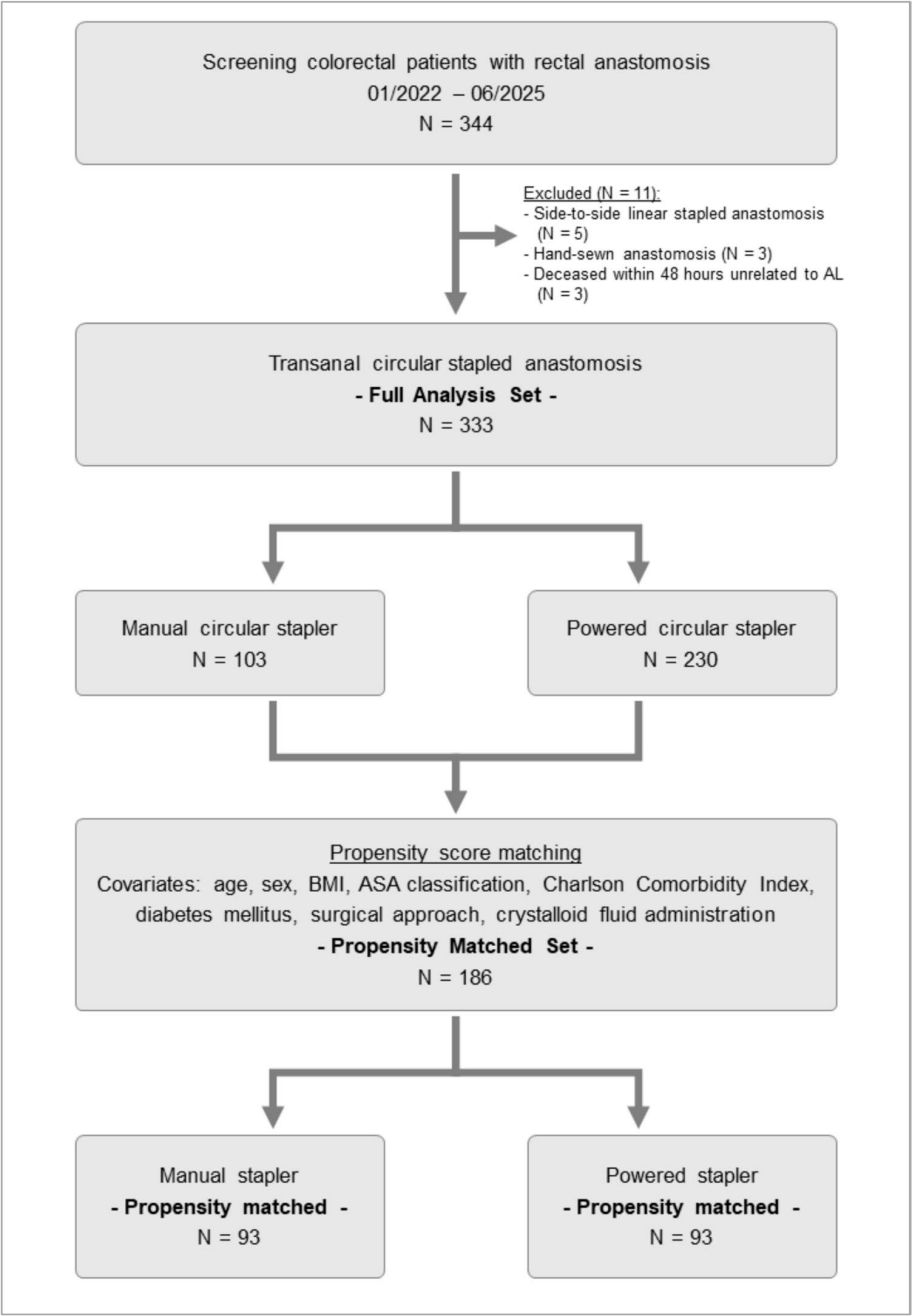
Flowchart of patient selection and propensity score matching. Flowchart illustrating consecutive patient selection, patient exclusions, distribution of patients between the manual and powered stapler groups, and the covariates used for propensity score matching. ASA classification = American Society of Anesthesiologists classification, BMI = body mass index

From January 2022 to June 2025, 344 colorectal patients with rectal anastomosis were screened. 11 patients were excluded, including five cases of side-to-side linear stapled anastomosis and three cases of hand-sewn anastomosis. Furthermore, three patients were excluded because they died within 48 h of surgery, precluding meaningful assessment of anastomotic healing. All three patients underwent open emergency surgery for perforated diverticulitis. Two patients (one manual stapler, one powered stapler) presented with severe septic shock and did not recover from the initial septic insult. The third patient (manual stapler) required prehospital cardiopulmonary resuscitation and subsequently developed brain death due to hypoxic brain injury, leading to withdrawal of life-sustaining therapy. A flowchart of patient selection is depicted in Fig. 1.

333 patients received colorectal resection with a primary transanal circular stapled anastomosis at our institution. Detailed patient characteristics of the full analysis set are provided in Table 1. AL was observed in 50 patients (15.0%) in total. Mean patient age was 61.1 years (95% CI 59.7—62.5), 183 patients were male (55.0%) and colon cases were more common than rectal (71.8 vs. 28.2%).

**Table 1:** Demographic characteristics of the Full Analysis Set

	Full Analysis Set (*N* = 333)	Full Analysis Set		
		Anastomotic leakage (*N* = 50)	No anastomotic leakage (N = 283)	*P value*
Age *[y] avg (95%CI)*	61.1 (59.7–62.5)	61.5 (58.0–65.0)	61.0 (59.5–62.6)	*.810*
Sex *n (%)*				
male	183 (55.0)	30 (60.0)	153 (54.1)	*.437*
female	150 (45.0)	20 (40.0)	130 (45.9)	
BMI *[kg/m*^*2*^*] avg (95%CI)*	26.8 (26.3–27.4)	28.1 (26.7–29.6)	26.6 (26.0–27.3)	***.021***
Data missing	6	0	6	
Type of case *n (%)*				
Colorectal	239 (71.8)	38 (76.0)	201 (71.0)	*.471*
Rectum	94 (28.2)	12 (24.0)	82 (29.0)	
Smoking *n (%)*	50 (15.0)	6 (12.8)	44 (16.7)	*.502*
Data missing	22	3	19	
Alcohol consumption *n (%)*	48 (14.4)	8 (17.4)	40 (15.5)	*.746*
Data missing	29	4	25	
ASA classification *n (%)*				
ASA < 3	165 (49.4)	18 (36.0)	147 (54.0)	***.019***
ASA ≥ 3	157 (47.1)	32 (64.0)	125 (46.0)	
Data missing	11			
Charlson Comorbidity Index *avg (95%CI)*	3.37 (3.08–3.65)	3.64 (2.80–4.48)	3.32 (3.02–3.62)	*.645*
Diabetes mellitus *n (%)*	35 (10.5)	12 (24.0)	23 (8.1)	** < *.001***
Immunosuppression *n (%)*	6 (1.8)	2 (4.0)	4 (1.4)	*.210*

ASA classification = American Society of Anesthesiologists classification, BMI = body mass index

Clinical characteristics that correlated significantly with the development of AL were higher BMI (AL: 28.1 vs. no AL: 26.6 kg/m^2^; p = 0.021), ASA ≥ 3 (64.0 vs. 46.0%; p = 0.019) and diabetes mellitus (24.0 vs. 8.1%; p < 0.001). Other known risk factors for AL, such as male gender, smoking or alcohol use, or immunosuppressive medication, did not show significant differences between patients with and without AL in our cohort.

### Surgical details

Surgical details are presented in Table 2. 230 patients (69.1%) received transanal circular anastomosis by a powered stapling device and 103 patients (30.9%) received manually stapled anastomosis.

**Table 2:** Surgical details of the Full Analysis Set

	Full Analysis Set (*N* = 333)	Full Analysis Set		
		Anastomotic leakage (*N* = 50)	No anastomotic leakage (*N* = 283)	*P value*
Stapling technique* n (%)*				
Powered stapler	230 (69.1)	38 (76.0)	192 (67.8)	*.250*
Manual stapler	103 (30.9)	12 (24.0)	91 (32.2)	
Indication *n (%)*				
Cancer	163 (48.9)	30 (60.0)	133 (47.0)	*.485*
Inflammation	129 (38.7)	15 (30.0)	114 (40.0)	
Perforation	28 (8.4)	4 (8.0)	24 (8.5)	
Intestinal obstruction	4 (1.2)	0	4 (1.4)	
Other	9 (2.7)	1 (2.0)	8 (2.8)	
Emergency surgery *n (%)*	56 (16.8)	11 (22.0)	45 (15.9)	*.288*
Surgical approach *n (%)*				
Robotic	180 (54.1)	19 (38.0)	161 (56.9)	***.004***
Laparoscopic	61 (18.3)	7 (14.0)	54 (19.1)	
Open	71 (21.3)	17 (34.0)	54 (19.1)	
Conversion to open	21 (6.3)	7 (14.0)	14 (4.9)	
Prior abdominal surgery *n (%)*	175 (52.6)	30 (61.2)	145 (51.4)	*.441*
Operation *n (%)*				
Colectomy	4 (1.2)	0	4 (1.4)	*.456*
Left/extended left hemicolectomy	130 (39.0)	24 (48.0)	106 (37.5)	
Rectosigmoid resection	132 (39.6)	18 (36.0)	114 (40.3)	
Low anterior rectal resection	67 (20.1)	8 (16.0)	59 (20.8)	
Stapler diameter* [mm] n (%)*				
28	16 (4.8)	1 (2.0)	15 (5.3)	*.690*
29	310 (93.1)	48 (96.0)	262 (92.6)	
31	5 (1.5)	1 (2.0)	4 (1.4)	
32	2 (.6)	0	2 (.7)	
Test for leakage *n (%)*	328 (98.5)	49 (98.0)	279 (98.6)	*.559*
Overstitching of stapler line *n (%)*	15 (4.5)	2 (4.0)	13 (4.6)	*1.0*
Prophylactic pelvic drain *n (%)*	66 (19.8)	11 (22.0)	55 (19.4)	*.675*
Diverting ostomy *n (%)*	104 (31.2)	14 (28.0)	90 (31.8)	*.593*
Duration of surgery *[min] avg (95%CI)*	200.0 (191.3–208.8)	199.6 (179.4–219.8)	200.1 (190.4–209.8)	*.841*
Intraoperative crystalloid fluid administration *[ml] avg (95%CI)*	2243.4 (2116.0–2370.9)	2548.1 (2193.8–2902.4)	2188.8 (2052.7–2325.0)	***.015***
Intraoperative colloids *n (%)*	36 (10.8)	6 (12.0)	30 (10.6)	*.775*
Intraoperative blood transfusion *n (%)*	22 (6.6)	5 (10.0)	17 (6.0)	*.298*
FAST TRACK *n (%)*	240 (72.1)	33 (66.0)	207 (73.1)	*.299*

The most common indications for surgery were cancer resection (163 patients, 48.9%) and inflammation (129 patients, 38.7%). Overall, 16.8% of procedures were performed in an emergency setting. The predominant surgical approach was robotic (180 cases, 54.1%), followed by open (71 cases, 21.3%) and laparoscopic surgery (61 cases, 18.3%). In 21 cases (6.3%), a minimally invasive procedure was converted to open surgery. The most frequent operations were left or extended left hemicolectomy (39.0%) and rectosigmoid resection (39.6%), followed by low anterior rectal resection (20.1%). An intraoperative leak test after stapling the anastomosis was performed in most patients (328, 98.5%), and overstitching of the stapler line was required in 15 cases (4.5%). Placement of a prophylactic pelvic drain and creation of a diverting ostomy were determined intraoperatively at the surgeon’s discretion and were performed in 66 (19.8%) and 104 (31.2%) patients, respectively. 72.1% patients followed the FAST TRACK protocol perioperatively.

AL occurred in 18.5% (24/130 cases) after left or extended left hemicolectomy, in 13.6% (18/132 cases) after rectosigmoid resection and in 11.9% (8/67 cases) after low anterior rectal resection (p = 0.456) with no statistically significant differences within the powered (20.0%/16.2%/13.0%) and the manual group (16.4%/6.1%/7.7%; p = 0.250). Neither neoadjuvant therapy nor tumor stage had a significant influence on the occurrence of AL. Among the 163 patients operated on for colorectal cancer, AL occurred in 22 of 108 patients without neoadjuvant treatment (20.4%) and in 8 of 54 patients who received neoadjuvant therapy (14.8%; p = 0.520; data missing for one patient). Likewise, no significant association was observed between tumor stage and AL occurrence (T0: 0/7, 0%; T1: 6/25, 24.0%; T2: 7/32, 21.9%; T3: 12/78, 15.4%; T4: 4/18, 22.2%; p = 0.631; data missing for three patients).

AL was associated with higher proportions of open (34.0 vs. 19.1%) or converted to open (14.0 vs. 4.9%; p = 0.004) surgery as well as greater intraoperative crystalloid fluid administration (2548.1 vs. 2188.8 ml; p = 0.015). Patients who developed an AL were slightly more likely to have undergone emergency surgery (22.0 vs. 15.9%; p = 0.288) and had marginally higher rates of intraoperative blood transfusion (10.0 vs. 6.0%; p = 0.298). Additionally, adherence to the FAST TRACK protocol (see Supplementary Table S2) was somewhat lower in the AL group (66.0 vs. 73.1%; p = 0.299). Notably, stapler diameter, overstitching of the stapler line, placement of a prophylactic pelvic drain and creation of a primary diverting ostomy did not correlate with AL development in our cohort.

### Multivariate analysis

Multivariable logistic regression analysis was performed to further assess risk factors for AL using all variable that revealed significant differences for AL occurrence in univariate analysis as well as known clinically relevant factors. The following variables were included: age, sex, BMI, ASA classification, Charlson Comorbidity Index, diabetes mellitus, surgical approach, operation and intraoperative crystalloid fluid administration. In multivariable logistic regression analysis, diabetes mellitus was independently associated with the occurrence of AL (OR 3.16, 95% CI 1.179–8.469, p = 0.022).

Of note, surgical approach showed a trend towards an association with the outcome in the multivariable analysis (overall p = 0.068). Therefore, adequate balancing of this variable was considered particularly important. Before propensity score matching, substantial imbalances were observed between groups with regard to the surgical approach (e.g., SMDs of 0.38 for robotic and 0.35 for laparoscopic procedures). After matching, these differences were markedly reduced (SMDs of 0.12 and 0.04, respectively), indicating successful balancing of one of the most relevant potential confounders.

### Postoperative complications

To isolate the effect of the powered versus manual stapling device on postoperative outcomes, patients were propensity score matched using the covariates age, sex and Charlson Comorbidity Index as well as all factors significantly associated with AL in our univariate analyses (BMI, ASA classification, diabetes mellitus, surgical approach and intraoperative fluid administration) (Fig. 1). A total of 93 patients who received a powered circular stapled anastomosis were matched to 93 patients who received a manually stapled anastomosis. Patient characteristics and surgical details before and after propensity score matching are summarised in Supplementary Table S3.

Overall, 81 of the 186 matched patients (43.6%) experienced any postoperative complication. Details on postoperative complications are provided in Table 3. The overall Comprehensive Complication Index (CCI) was 16.5 (95%CI 13.0—19.9), with not differences between the manual (17.0, 95%CI 12.1–21.9) and the powered stapling device (15.9, 95%CI 10.9–20.9; p = 0.869). AL occurred in 20 patients (10.8%) overall with 11.8% (11/93 patients) in the manual stapler and 9.7% (9/93 patients) in the powered stapler cohort (p = 0.636).

**Table 3:** Postoperative complications of the Propensity Matched Set

	Propensity Matched Set (*N* = 186)	Propensity Matched Set		
		Manual stapler (*N* = 93)	Powered stapler (*N* = 93)	*P value*
CCI *avg (95%CI)*	16.5 (13.0–19.9)	17.0 (12.1–21.9)	15.9 (10.9–20.9)	*.869*
Anastomotic leakage* n (%)*	20 (10.8)	11 (11.8)	9 (9.7)	*.636*
Superficial surgical site infection *n (%)*	21 (11.3)	10 (10.8)	11 (11.8)	*.817*
Pneumonia* n (%)*	6 (3.2)	2 (2.2)	4 (4.3)	*.682*
Cardial complication* n (%)*	4 (2.2)	1 (1.1)	3 (3.2)	*.621*
Thromboembolic complication* n (%)*	1 (.5)	0	1 (1.1)	*1.0*
Urinary tract infection* n (%)*	6 (3.2)	2 (2.2)	4 (4.3)	*.682*
Delirium* n (%)*	6 (3.2)	5 (5.4)	1 (1.1)	*.211*
Length of stay [d] *avg (95%CI)*	9.77 (8.58–10.97)	9.94 (8.10–11.79)	9.60 (8.05–11.15)	*.467*
Mortality 90 days *n (%)*	2 (1.1)	1 (1.1)	1 (1.1)	*1.0*

*CCI* = Comprehensive Complication Index

The most frequently observed additional complications was superficial site infection (21 patients, 11.3%). Hospital length of stay was 9.8 days (95%CI 8.6—11.0 days) on average with no significant difference between the manual (9.9 days) and the powered stapling group (9.6 days, p = 0.467). Two patients died during postoperative recovery, with no differences between the two stapling devices. One patient (manual stapling group) died after prolonged intensive care treatment and multiple reoperations due to recurrent stoma detachments and extensive wound complications, which resulted in an abdominal wall phlegmon and septic decompensation. The other patient (powered stapling group) experienced a cardiac event and did not recover from succeeding cardiogenic shock.

Since the powered stapling technique did not affect the occurrence of AL, we subsequently analysed the severity and management of AL. Details related to AL are summarised in Table 4. On average, AL was diagnosed after 5.8 days (95%CI 4.55—7.05). Most patients required reoperation to manage AL: 10 patients (50.0%) were treated surgically alone, four patients (20.0%) underwent reoperation combined with endoscopic vacuum therapy (EVT), three patients (15.0%) received EVT alone and three patients (15.0%) were managed with antibiotics only. The reoperation rate did not differ between the manual (63.6%, 7/11) and powered stapling cohort (77.8%, 7/9; p = 0.642).

**Table 4:** Anastomotic leakage severity and management of the Propensity Matched Set

	Propensity Matched Set (*N* = 20)	Propensity Matched Set		
		Manual stapler (*N* = 11)	Powered stapler (*N* = 9)	*P value*
Time from surgery to AL diagnosis *[d] avg (95%CI)*	5.80 (4.55–7.05)	5.91 (4.10–7.72)	5.67 (3.53–7.81)	*.846*
CDC related to AL *n (%)*				
CDC I + II	0	0	0	*.638*
CDC IIIa	6 (30.0)	4 (36.4)	2 (22.2)	
CDC IIIb	11 (55.0)	6 (54.5)	5 (55.6)	
CDC IVa	3 (15.0)	1 (9.1)	2 (22.2)	
CDC IVb	0	0	0	
CDC V	0	0	0	
AL management *n (%)*				*.367*
Antibiotics only	3 (15.0)	1 (9.1)	2 (22.2)	
EVT	3 (15.0)	3 (27.3)	0	
Reoperation and EVT combined	4 (20.0)	2 (18.2)	2 (22.2)	
Reoperation	10 (50.0)	5 (45.5)	5 (55.6)	

*AL* = anastomotic leakage, *CDC* = Clavien Dindo Classification, *EVT* = endoscopic vacuum therapy

In the manual stapling group, seven patients required reoperation. Indications included recreation of the anastomosis in six patients, of whom four simultaneously received a diverting ostomy, while one patient underwent diverting ostomy creation alone. Two patients who underwent diverting ostomy creation – one in combination with anastomotic recreation and the one as a standalone procedure – also received adjunctive EVT. Overall, three patients underwent relaparotomy, one patient required secondary conversion to open surgery and three patients were managed by relaparoscopy.

In the powered stapling group, six patients underwent diverting ostomy creation. Of these, two patients simultaneously underwent anastomotic recreation, two received anastomotic overstitching and two were treated with adjunctive EVT. One patient required Hartmann’s procedure due to septic shock.

No significant differences were observed between the powered and the manual stapling groups regarding time to AL diagnosis, AL-related Clavien-Dindo classification or AL management strategies. No patient in the study cohort died because of AL.

### Material costs

The costs for the different products were higher for powered staplers (607€ Ethicon, Hamburg, Germany) compared to manually operated staplers (330€ (Covidien, Dublin, Ireland and 240€ Frankenman, Kiel, Germany).

## Discussion

The aim of this study was to evaluate if the type of transanal circular stapling device—powered vs. manual—impacts AL development after colorectal resections. Our results suggest that the performance of the powered stapler is not superior to manual circular staplers concerning the development of postoperative complications and AL in particular.

The overall incidence of AL in our cohort was 15.0%, which is comparable to AL rates reported after colorectal resections in the current literature [1, 4]. We identified several patient-related risk factors for the development of AL that have been described previously, including ASA classification, BMI and diabetes mellitus [1, 3, 10]. While these factors are clinically relevant, they are only partially or not readily modifiable in the perioperative setting. Other patient-related variables that are known to influence AL, such as age and male sex [1, 3], did not significantly influence AL rates in our cohort.

In contrast, our analysis placed particular emphasis on potentially modifiable, surgery-related factors that were associated with the development of AL. These factors include surgical approach and the amount of intraoperative crystalloid fluid administration, both of which have been investigated previously and may present targets for perioperative optimisation.

In our cohort, robotic and laparoscopic colorectal resections were associated with the lowest observed AL rates (10.6 and 11.5%) compared to open surgery (23.9%) and conversions to open surgery (33.3%). The influence of surgical approach on AL risk has been explored in large retrospective cohorts and systematic analyses [3]. For example, Murray et al. found that laparoscopic colorectal surgery was associated with a significantly reduced AL rate compared to open surgery in a large propensity score-adjusted cohort, supporting an independent effect of surgical approach on AL risk [19]. Some studies suggest lower AL rates for robotic surgery compared to laparoscopic approaches, although results remain inconsistent across different cohorts and procedure types [20]. Overall, existing data suggest that minimally invasive approaches (laparoscopic and robotic) may be associated with lower AL rates than open surgery, although evidence remains heterogeneous and further robust comparative studies are required.

In contrast, intraoperative fluid administration represents a potentially modifiable but underreported risk factor for AL, with existing evidence remaining heterogeneous. Van Rooijen et al. identified intraoperative fluid management as a potentially modifiable factor affecting anastomotic integrity but did not demonstrate a direct association between fluid volume and AL [21]. In contrast, a systematic review and meta-analysis by Jessen et al. showed that goal-directed hemodynamic therapy during noncardiac surgery may reduce postoperative complications including the development of AL [22]. Our findings are in line with these observations: higher intraoperative crystalloid fluids were associated with AL (2548 vs. 2189 ml).

However, fluid volumes may also reflect more complex procedures or septic presentations. These findings support goal-directed intraoperative fluid management, particularly in elective surgeries. For emergency surgeries this aspect cannot be answered from the data.

Several previously reported surgery-related risk factors, including prophylactic pelvic drainage, emergency surgery and diverting ostomy creation [3, 4, 23], were not associated with AL in our cohort.

Furthermore, the type of operation and thus the level of the transanal anastomosis was not significantly associated with AL development. Previous studies have consistently reported the highest AL rates following low anterior rectal resection requiring low-level anastomosis, with leakage rates of up to 19.0% reported in the literature [1, 3, 4]. By comparison, in our cohort, left or extended left hemicolectomy was associated with the highest AL rate (18.5%), whereas AL occurred in only 11.9% of patients undergoing low anterior resections. This difference might be due to less protective ostomies in this group compared to the low anterior resections, in which smaller leakages may presented only with mild symptoms and may have not been detected whereas left hemicolectomies without ostomy did develop more severe clinical symptoms. Another factor may have been the surgeon’s experience, since low anterior resections have been performed by the most experienced and well-trained colorectal surgeons in our department.

Leakage management in our institution is increasingly based on endoscopic vacuum therapy (EVT) [24]. In our cohort no image-guided drainages were performed, despite this technique belongs to our portfolio. This reflects both our preference for EVT and the limited accessibility of pelvic collections. But this approach may also be suitable if sufficient expertise is given.

After identifying several AL-related risk factors in univariate analysis, we performed propensity score matching to isolate the impact of the transanal circular stapling device (powered vs. manual) on clinical outcomes. To generate comparable cohorts, matching was conducted based on age, sex, BMI, ASA classification, Charlson Comorbidity Index, diabetes mellitus, surgical approach and intraoperative crystalloid fluid administration. After matching, overall CCI as well as the incidence of common postoperative complications following colorectal resection, including surgical site infections, were comparable between the two groups.

Following propensity score matching, AL rates were similar between the stapling groups, with an incidence of 11.8% in the manual stapling group and 9.7% in the powered stapling group. Likewise, AL-related CDC did not differ between groups. Management of AL was also comparable, with similar rates of reoperation (manual: 63.6 vs. powered: 77.8%). These findings do not support previous reports suggesting lower AL rates or reduced reoperation rates following powered circular stapled anastomoses.

Three meta-analyses have suggested that powered staplers may reduce AL, but the underlying evidence requires critical interpretation. In the meta-analysis by Fiorello et al., six retrospective studies compared powered and manual staplers, with four reporting lower AL rates with powered devices [25]. Three of these four studies disclosed consultancy relationships with the manufacturer of the powered stapler [10, 11, 14] and two reported exceptionally low AL rates (1.7—1.8%), raising concerns regarding external validity [10, 14]. In contrast, two independent retrospective analyses observed no significant difference in AL rates between powered and manual staplers [12, 15].

Of the three independent analyses, only one favoured powered staplers [13]. This study included a relatively small cohort of 187 patients over a 6.5-year period (01/2016—07/2022) and likely compared powered staplers introduced in 2019 with a historical manual stapler cohort. The manual group contained more open procedures, whereas the powered group had a higher rate of robot-assisted surgery, even after propensity score matching. These potential confounders, together with changes in perioperative care such as FAST TRACK implementation, were not adequately addressed. Similar limitations were present in another industry-funded study using a historical control cohort with higher conversion rates [14]. Therefore, the conclusions of this meta-analysis must be interpreted with caution.

The meta-analysis by Ohtani et al. incorporated the same six studies plus three additional retrospective analyses [26]. One additional study declared a conflict of interest (affiliation with the powered stapler manufacturer), whereas the other two reported no significant differences in AL rates [8, 9, 27]. Nevertheless, pooled analysis favoured powered staplers. Overall, four of the five studies reporting benefit for powered staplers were associated with the manufacturer, whereas most independent studies observed no advantage.

The most recent meta-analysis by Martín-Arévalo et al. added three further retrospective studies. One found no difference between stapler types, one reported higher leakage rates with powered staplers and one favoured powered staplers [28]. Although the authors reported no conflicts of interest in the meta-analysis, previous publications by the senior author consistently disclosed consultancy fees from the powered stapler manufacturer [10, 29].

Importantly, all studies included in both meta-analyses were retrospective in nature. In contrast, a recent independently conducted prospective randomized controlled trial found no significant differences in AL rates between powered and manual staplers, although a modest reduction in anastomosis configuration time was reported (powered: 2.4 vs. manual: 2.8 min) [30]. Whether this time difference of approximately 0.4 min justifies the increased cost of powered staplers in the absence of improved anastomotic healing remains a matter of clinical judgement.

Furthermore, to our knowledge none of these retrospective studies mentioned above that were included in the two meta-analyses had a defined postoperative protocol to evaluate possible AL as reported in our study protocol with a defined diagnostic algorithm (see Supplementary Figure S1).

This study has several limitations related to its retrospective single-centre design. Selection bias cannot be completely excluded, as factors such as stapler choice and surgical approach may have been influenced by technical considerations. Additionally, AL detection rates may have been influenced by ostomy creation as already discussed above. Furthermore, exclusion of patients who died in the immediate postoperative period, in whom anastomotic assessment was not feasible, may limit the generalizability of our findings to emergency surgery settings.

We tried to rule out the comparison with historical cohort with mainly open surgery before implementation of FAST TACK concepts to minimize these influences and a very strict matching caliper. This has led to a smaller sample size, but a better data quality and better comparability. The reduced sample size after strict matching may have limited the statistical power to detect small differences between stapler types. Regarding the results of a ~ 20% effect size between the groups a sample size of > 560 cases per group would have been necessary to reveal a possible statistical significance.

In summary, the potential benefit of powered circular staplers for transanal anastomosis remains unclear based on the currently available evidence. Powered circular staplers may offer possible technical advantages, including more uniform force application during firing, increased motion stability and a modest reduction in anastomosis firing time [30]. However, robust evidence demonstrating a clinically relevant advantage for patients is currently lacking. Further independent, prospective studies are required to determine whether powered circular staplers provide patient-relevant benefits that would justify their increased costs compared with conventional manual stapling devices.

## Conclusion

In this retrospective cohort study conducted under contemporary perioperative FAST TRACK protocol and minimally invasive surgery conditions, powered circular stapling devices were not superior to manual staplers for transanal colorectal anastomosis. While higher ASA-classification, BMI and diabetes mellitus were confirmed as patient-related risk factors for AL development, these factors are only partially modifiable, underscoring the importance of optimising potentially modifiable, risk-reducing surgical and perioperative factors such as surgical approach and intraoperative fluid management. After adjustment for relevant patient- and surgery-related risk factors using propensity score matching, no significant differences were observed between stapler types with respect to AL rate, leakage severity or management strategies.

Overall, our findings do not support a clinically relevant advantage of powered circular staplers over manual devices in terms of anastomotic healing. Further independent, prospective and adequately designed studies are required to determine whether the potential technical advantages of powered stapler translate into relevant patient benefit.

## Supplementary Information

Below is the link to the electronic supplementary material.

Supplementary file1 (PDF 170 KB)

Supplementary file2 (PDF 109 KB)

Supplementary file3 (PDF 162 KB)

Supplementary file4 (PDF 105 KB)

Supplementary file5 (PDF 141 KB)

## Data Availability

The data that support the findings of this study are available from the corresponding author upon reasonable request.
