# Supplementary material for: Impact of powered circular stapling devices on anastomotic leakage rates in colorectal surgery
Source: Int J Colorectal Dis. 2026 Jul 8;41(1):114. doi: 10.1007/s00384-026-05195-7 (PMC13346286; doi:10.1007/s00384-026-05195-7)
Supplement: Supplementary file 2 — Supplementary file2 (PDF 109 KB) [file 384_2026_5195_MOESM2_ESM.pdf]

Manuscript Title
Impact of powered circular stapling devices on anastomotic leakage rates in colorectal surgery
Journal
International Journal of Colorectal Disease
Authors
Catherine Kollmann, Theresa Eckart, Beata Kusnezov, Lars Kollmann, Matthias Kelm, Christoph-Thomas Germer, Johan Friso Lock, Sven Flemming*
***Corresponding author:**
PD Dr. med. Sven Flemming
Department of General, Visceral, Transplant, Vascular and Paediatric Surgery, University Hospital Würzburg
Email: Flemming_S@ukw.de

Supplementary Table S2 Institutional FAST TRACK protocol for colorectal surgeries

**Preoperative items**	Patient information by specialized FAST TRACK assistants
	Optimisation of patient’s health status including prehabilitation
	Nutritional risk assessment and therapy of malnutrition
	Anemia screening and management
	No mechanical bowel preparation (only oral antibiotics on the day before surgery)
	No preoperative fasting
	Carbohydrate drinks
	Euvolemia
	Prevention of postoperative nausea and vomiting
	No anxiolytic premedication (no benzodiazepines)
	Hospital admission on day of surgery
**Intraoperative items**	Prophylactic intravenous antibiotics before skin incision
	Standard anesthesia protocol
	Normovolemia and normothermia
	Adequate regional analgesia (EDA in open surgery, TAP-block in minimal invasive surgery)
	Minimal invasive surgery if possible
	No drainage
**Postoperative items**	No gastric tube
	Multimodal oral analgesia
	Thromboprophylaxis
	Normovolemia
	Removal of urinary catheter on POD 1
	Immediate oral nutrition (including high-protein drinks)
	Early mobilisation starting on day of surgery (POD 0: > 15 min, POD 1: > 4 h, POD 2: > 6 h, POD 3: > 8 h)

EDA = epidural anesthesia, POD = postoperative day, TAP = transversus abdominis plane
