# Supplementary material for: Impact of powered circular stapling devices on anastomotic leakage rates in colorectal surgery
Source: Int J Colorectal Dis. 2026 Jul 8;41(1):114. doi: 10.1007/s00384-026-05195-7 (PMC13346286; doi:10.1007/s00384-026-05195-7)
Supplement: Supplementary file 3 — Supplementary file3 (PDF 162 KB) [file 384_2026_5195_MOESM3_ESM.pdf]

Manuscript Title
Impact of powered circular stapling devices on anastomotic leakage rates in colorectal surgery
Journal
International Journal of Colorectal Disease
Authors
Catherine Kollmann, Theresa Eckart, Beata Kusnezov, Lars Kollmann, Matthias Kelm, Christoph-Thomas Germer, Johan Friso Lock, Sven Flemming*
***Corresponding author:**
PD Dr. med. Sven Flemming
Department of General, Visceral, Transplant, Vascular and Paediatric Surgery, University Hospital Würzburg
Email: Flemming_S@ukw.de

Supplementary Table S3 Demographics and surgical details before and after propensity score matching

	**Pre-propensity score matching**			**Post-propensity score matching**		
	Manual stapler (N = 103)	Powered stapler (N = 230)	P value	Manual stapler (N = 93)	Powered stapler (N = 93)	P value
**Age [y] avg (95%CI)**	60.44 (57.7-63.4)	61.4 (59.7-63.1)	.537	61.1 (58.3-63.9)	60.6 (57.9-63.3)	.823
**Sex n (%)**						
**male**	52 (50.5)	131 (57.0)	.273	47 (50.5)	41 (44.1)	.378
**female**	51 (49.5)	99 (43.0)		46 (49.5)	52 (55.9)	
**BMI [kg/m^2] avg (95%CI)**	26.4 (25.5-27.3)	26.9 (26.2-27.7)	.698	26.2 (25.0-27.1)	25.1 (24.3-26.0)	.104
**Smoking n (%)**	14 (15.1)	36 (16.5)	.748	11 (12.8)	14 (15.9)	.558
**Alcohol consumption n (%)**	16 (18.2)	32 (14.8)	.465	16 (19.3)	10 (11.5)	.159
**ASA classification n (%)**						
**ASA <3**	59 (60.8)	106 (47.1)	**.024**	58 (62.4)	61 (65.6)	.647
**ASA ≥3**	38 (39.2)	119 (52.9)		35 (37.6)	32(34.4)	
**Charlson Comorbidity Index avg (95%CI)**	2.70 (2.24-3.16)	3.67 (3.32-4.01)	**.002**	2.82 (2.33-3.30)	2.98 (2.46-3.49)	.853
**Diabetes mellitus n (%)**	7 (6.8)	28 (12.2)	.139	7 (7.5)	3 (3.2)	.193
**Indication n (%)**						
**Cancer**	48 (46.6)	115 (50.0)	.279	45 (48.5)	45 (48.5)	.666
**Inflammation**	41 (39.8)	88 (38.3)		37 (39.8)	42 (45.2)	
**Perforation**	7 (6.8)	21 (9.1)		6 (6.5)	4 (4.3)	
**Intestinal obstruction**	3 (2.9)	1 (0.4)		1 (1.1)	0	
**Other**	4 (3.9)	5 (2.2)		4 (4.3)	4 (4.3)	
**Emergency surgery n (%)**	23 (22.3)	33 (14.3)	.072	17 (18.3)	8 (8.6)	.053
**Surgical approach n (%)**						
**Robotic**	69 (67.0)	111 (48.3)	**.008**	65 (59.9)	70 (75.3)	.714
**Laparoscopic**	10 (9.7)	51 (22.2)		9 (9.7)	10 (10.8)	
**Open**	19 (18.4)	52 (22.6)		16 (17.2)	11 (11.8)	
**Conversion to open**	5 (4.9)	16 (7.0)		3 (3.2)	2 (2.2)	
**Prior abdominal surgery n (%)**	53 (51.5)	122 (53.5)	.321	46 (49.5)	47 (50.5)	.600
**Operation n (%)**						
**Colectomy**	2 (1.9)	2 (0.9)	**.002**	1 (1.1)	0	**.004**
**Left/extended left hemicolectomy**	55 (53.4)	75 (32.6)		50 (53.8)	27 (29.0)	
**Rectosigmoid resection**	33 (32.0)	99 (43.0)		29 (31.2)	42 (45.2)	

**Low anterior rectum resection**	13 (12.6)	54 (23.5)		13 (14.0)	24 (25.8)	
**Test for leakage n (%)**	101 (98.1)	227 (98.7)	.647	91 (97.8)	93 (100.0)	.155
**Overstitching of stapler line n (%)**	3 (2.9)	12 (5.2)	.349	3 (3.2)	3 (3.2)	1.0
**Prophylactic pelvic drain n (%)**	21 (20.4)	45 (19.6)	.862	16 (17.2)	10 (10.8)	.205
**Diverting ostomy n (%)**	29 (28.2)	75 (32.6)	.418	26 (28.0)	28 (30.1)	.747
**Duration of surgery [min] avg (95%CI)**	181.1 (170.2-192.0)	208.5 (196.9-220.1)	**.005**	178.5 (167.8-189.2)	211.5 (189.5-233.5)	**.017**
**Intraoperative crystalloid fluid administration [ml] avg (95%CI)**	1975.5 (1802.6-2148.4)	2363.8 (2197.9-2529.8)	**.003**	1938.1 (1775.6-2100.6)	1993.0 (1815.6-2170.4)	.548

ASA classification = American Society of Anesthesiologists classification, BMI = body mass index
