# Supplementary material for: Impact of powered circular stapling devices on anastomotic leakage rates in colorectal surgery
Source: Int J Colorectal Dis. 2026 Jul 8;41(1):114. doi: 10.1007/s00384-026-05195-7 (PMC13346286; doi:10.1007/s00384-026-05195-7)
Supplement: Supplementary file 4 — Supplementary file4 (PDF 105 KB) [file 384_2026_5195_MOESM4_ESM.pdf]

Manuscript Title
Impact of powered circular stapling devices on anastomotic leakage rates in colorectal surgery
Journal
International Journal of Colorectal Disease
Authors
Catherine Kollmann, Theresa Eckart, Beata Kusnezov, Lars Kollmann, Matthias Kelm, Christoph-Thomas Germer, Johan Friso Lock, Sven Flemming*
***Corresponding author:**
PD Dr. med. Sven Flemming
Department of General, Visceral, Transplant, Vascular and Paediatric Surgery, University Hospital Würzburg
Email: Flemming_S@ukw.de

Supplementary Fig. S1 Flowchart of diagnostic algorithm for anastomotic leakage

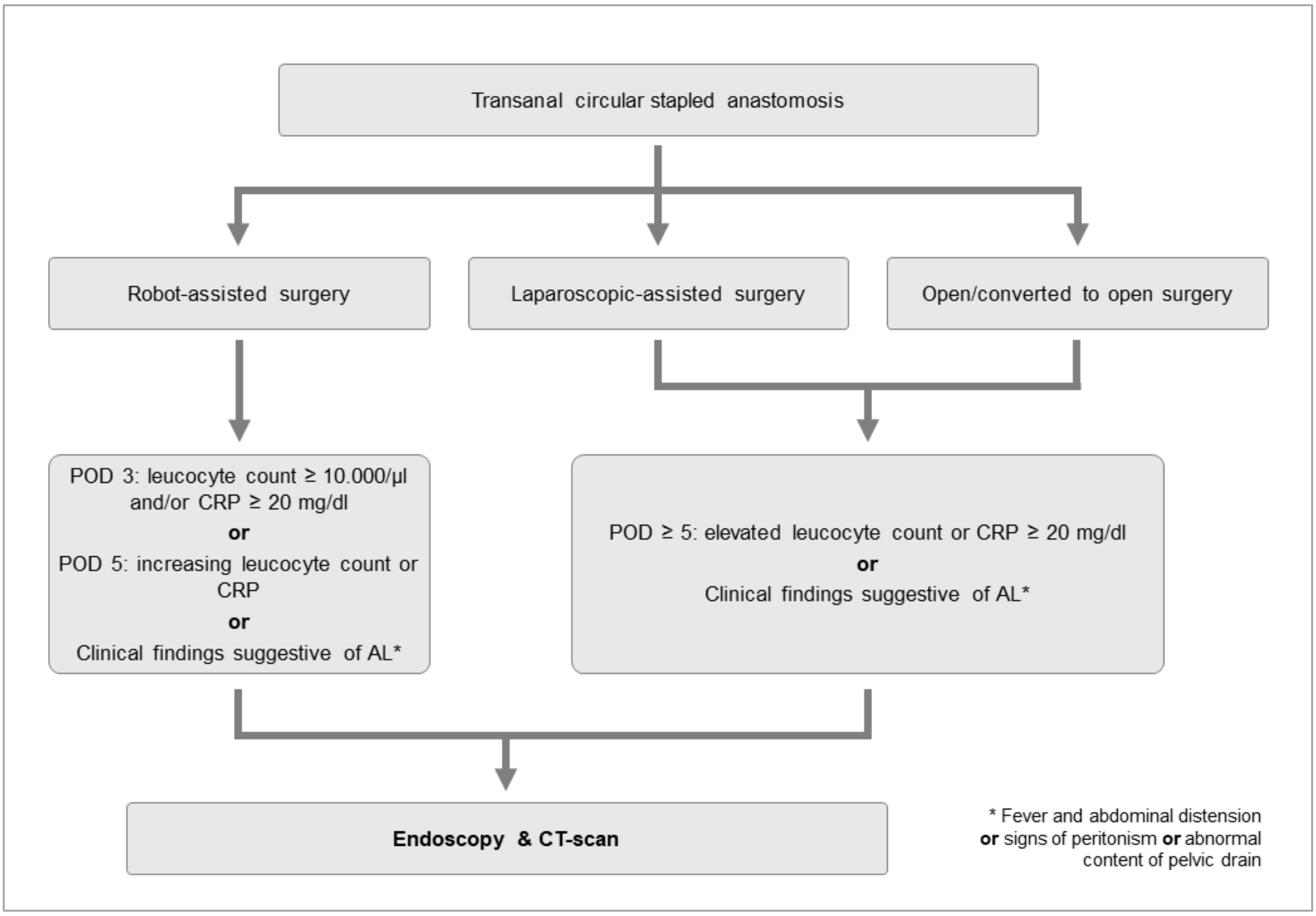

Flowchart of the diagnostic algorithm for the detection of anastomotic leakage, including the diagnostic pathways for different surgical approaches and the resulting diagnostic procedures

CRP = C-reactive protein, CT = computed tomography, POD = postoperative day
