# Supplementary material for: Impact of powered circular stapling devices on anastomotic leakage rates in colorectal surgery
Source: Int J Colorectal Dis. 2026 Jul 8;41(1):114. doi: 10.1007/s00384-026-05195-7 (PMC13346286; doi:10.1007/s00384-026-05195-7)
Supplement: Supplementary file 5 — Supplementary file5 (PDF 141 KB) [file 384_2026_5195_MOESM5_ESM.pdf]

Manuscript Title
Impact of powered circular stapling devices on anastomotic leakage rates in colorectal surgery
Journal
International Journal of Colorectal Disease
Authors
Catherine Kollmann, Theresa Eckart, Beata Kusnezov, Lars Kollmann, Matthias Kelm, Christoph-Thomas Germer, Johan Friso Lock, Sven Flemming*
***Corresponding author:**
PD Dr. med. Sven Flemming
Department of General, Visceral, Transplant, Vascular and Paediatric Surgery, University Hospital Würzburg
Email: Flemming_S@ukw.de

Supplementary Fig. S2 Love plot of standardised mean differences before and after Propensity score matching

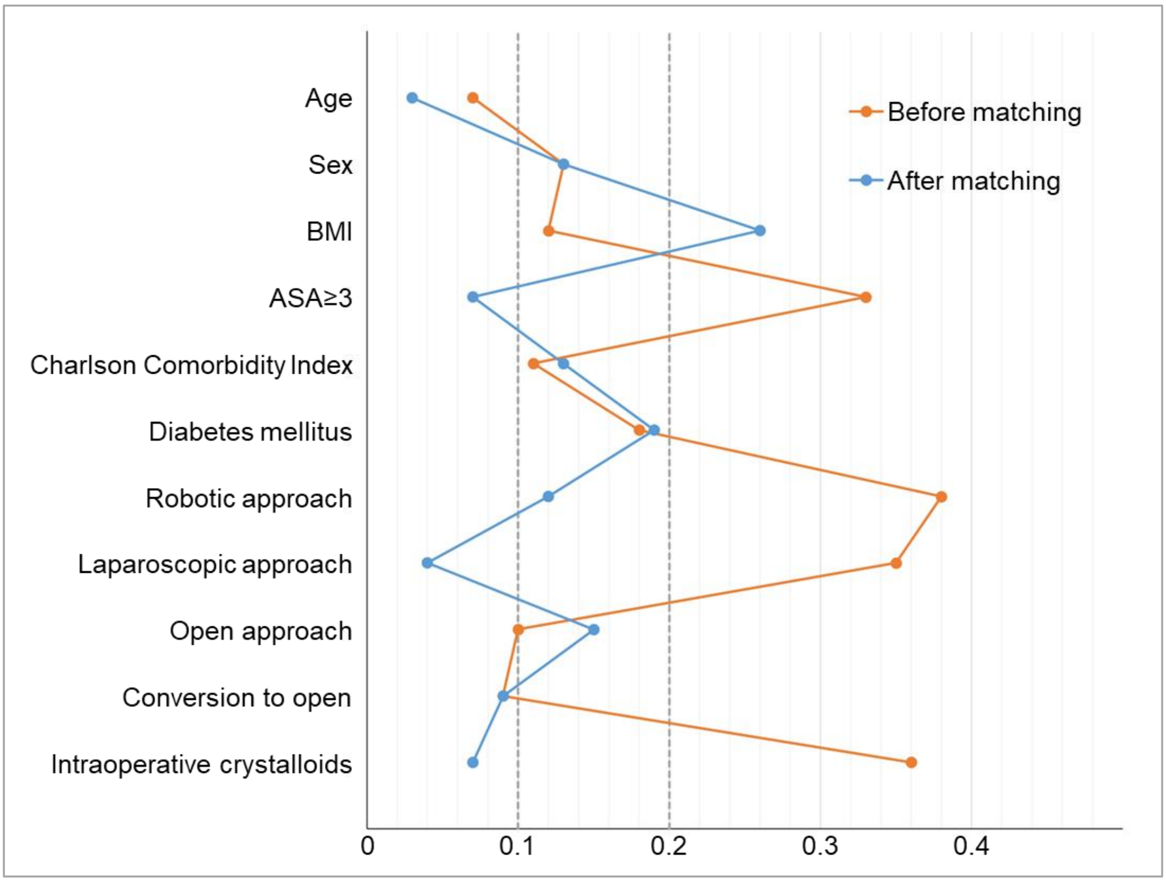

Love plot depicting standardised mean differences of relevant covariates before and after propensity score matching. Values before matching are shown in orange and values after matching in blue.

BMI = body mass index, ASA classification = American Society of Anesthesiologists classification
